# Nutritional Ketosis as a Potential Treatment for Alcohol Use Disorder

**DOI:** 10.3389/fpsyt.2021.781668

**Published:** 2021-11-30

**Authors:** Vikrant R. Mahajan, Sophie K. Elvig, Leandro F. Vendruscolo, George F. Koob, Valerie L. Darcey, M. Todd King, Henry R. Kranzler, Nora D. Volkow, Corinde E. Wiers

**Affiliations:** ^1^Department of Pharmacology, Vanderbilt University, Nashville, TN, United States; ^2^Integrative Neuroscience Research Branch, National Institute on Drug Abuse, Baltimore, MD, United States; ^3^National Institute of Diabetes and Digestive and Kidney Diseases, Bethesda, MD, United States; ^4^National Institute on Alcohol Abuse and Alcoholism, Rockville, MD, United States; ^5^Perelman School of Medicine, University of Pennsylvania, Philadelphia, PA, United States

**Keywords:** alcoholism, alcohol dependence, alcohol withdrawal, ketogenic diet, ketone ester, alcohol metabolism

## Abstract

Alcohol use disorder (AUD) is a chronic, relapsing brain disorder, characterized by compulsive alcohol seeking and disrupted brain function. In individuals with AUD, abstinence from alcohol often precipitates withdrawal symptoms than can be life threatening. Here, we review evidence for nutritional ketosis as a potential means to reduce withdrawal and alcohol craving. We also review the underlying mechanisms of action of ketosis. Several findings suggest that during alcohol intoxication there is a shift from glucose to acetate metabolism that is enhanced in individuals with AUD. During withdrawal, there is a decline in acetate levels that can result in an energy deficit and could contribute to neurotoxicity. A ketogenic diet or ingestion of a ketone ester elevates ketone bodies (acetoacetate, β-hydroxybutyrate and acetone) in plasma and brain, resulting in nutritional ketosis. These effects have been shown to reduce alcohol withdrawal symptoms, alcohol craving, and alcohol consumption in both preclinical and clinical studies. Thus, nutritional ketosis may represent a unique treatment option for AUD: namely, a nutritional intervention that could be used alone or to augment the effects of medications.

## Introduction

Alcohol use disorder (AUD) is a chronic, relapsing disorder characterized by disrupted function of brain circuits involved with reward, self-regulation, and emotion. During early abstinence or acute withdrawal, patients with AUD often exhibit signs and symptoms of the alcohol withdrawal syndrome (AWS), including intense alcohol craving, negative emotional states, restlessness, and in severe cases, seizures and delirium tremens. Treatment with benzodiazepines is currently the safest, most effective treatment for acute alcohol withdrawal, reducing the risk of serious symptoms such as seizures (1, 2). However, there is risk of dependence on benzodiazepines, particularly among patients with AUD, which precludes their use in this population beyond the period of acute withdrawal (3). Although anticonsulvants have also been shown to be efficacious in treating AWS and have less potential for dependence, these medications have a number of adverse effects (4, 5). Thus, additional efficacious treatments are needed that have less dependence potential and adverse effects than existing medications.

Recent preclinical and clinical studies show beneficial effects of a nutritional state of ketosis on alcohol withdrawal symptoms (6–8). Ketosis is characterized by elevated plasma and brain levels of ketone bodies (acetoacetate [AcAc], β-hydroxybutyrate [BHB] and acetone) that can be induced by prolonged or intermittent fasting, consumption of a low-carbohydrate, high-fat Ketogenic Diet (KD), a nutritional Ketone Ester (KE) supplement, Medium Chain Triglyceride (MCT) oils, or D-β-hydroxybutyrate (D-BHB) ketone salts. Here, we review the literature on the use of ketosis implemented using dietary interventions and the rationale for its potential use as a treatment for AUD. We also propose several mechanistic hypotheses based on the extant literature.

## Nutritional Ketosis

Nutritional ketosis is a physiological state of energy consumption that relies primarily on elevated concentrations of ketone bodies. Ketone body concentrations can be elevated indirectly through ketogenic diets and prolonged fasts to promote fatty acid catabolism or directly through dietary supplementation with D-BHB ketone salts or esters. In addition, MCTs offer another potential avenue for supplementation *via* octanoic and decanoic acids, which produce more ketones per unit of energy than dietary fat (9, 10). A KD with a traditional 4:1 ratio of grams of fat to grams of carbohydrates/protein (i.e., 80% calories from fat, 15% calories from protein and 5% calories from carbohydrates) raises blood BHB levels up to 4.5 mM (8), while D-BHB salts and MCT oils elevate peak blood levels of BHB to around 0.5 mM (11, 12) and the D-BHB ketone ester raises BHB levels to ~3.2 mM (13–15).

In the presence of insufficient carbohydrates, hepatic catabolism of fatty acids from triglycerides increases ketone body levels in plasma and brain, inducing a state of metabolic ketosis. A KD shifts energy metabolism toward β-oxidation, the mitochondrial aerobic catabolism of fatty acids into acetyl-CoA (16), which can reduce the risk of seizures in patients with epilepsy (17–21). In addition, KDs have shown therapeutic effects in patients with Alzheimer's disease (22) and Parkinson's disease (23), and have been proposed as a potential therapeutic intervention for psychiatric disorders such as autism spectrum disorder (24, 25), major depressive disorder (26, 27), schizophrenia (28, 29), and bipolar disorder (30, 31). However, patient adherence to KDs, particularly those that most tightly restrict carbohydrate content (32), is limited by their poor palatability.

The nutritional supplement (R)-3-hydroxybutyl (R)-3-hydroxybutyrate (Ketone Ester; KE) is a safe (13, 33), effective, and commercially available method (e.g., DeltaG^®^, TdeltaS^®^, Orlando FL) for inducing ketosis. KE has been shown to stabilize brain networks, thereby protecting the hypometabolic, aging brain (34), increasing physical endurance in athletes (35) and improving indices of cognition in preclinical and clinical models of Alzheimer's Disease (36–38). Several advantages of ketone supplementation, specifically with D-BHB, over the traditionally used KD have been described. Within 30 min of its administration, KE (which is commercially available in a slightly bitter but palatable liquid) induces levels of plasma ketone bodies similar to those observed after 2 weeks of KD, with the effects maintained for 4–5 h with no further dietary manipulation (8, 15). Although KEs anecdotally are more effective in fasted states, their use, in contrast to KDs, does not require drastic carbohydrate restriction (39). Finally, KEs directly increase plasma ketone body levels, circumventing potential alcohol-induced inhibition of AMP-activated protein kinase (AMPK), a master regulator of ketogenesis (40, 41).

## Effects of Nutritional Ketosis on Alcohol Withdrawal

Preclinical and clinical research provide evidence that KD-induced nutritional ketosis is a feasible strategy for mitigating the debilitating effects of alcohol withdrawal. Dencker et al. (6) measured the effect of a KD on signs of alcohol withdrawal in a rodent model of alcohol dependence. They found that, compared to regular chow, a KD attenuated muscular rigidity and irritability in alcohol-dependent rats during alcohol detoxification. However, despite previous evidence that exogenous ketone supplementation has anxiolytic effects in the elevated plus maze test (36, 42), this study showed no significant effect of the KD on anxiety-like behavior as measured either by the elevated plus maze test or locomotor activity (6). One potentially confounding factor in the study was that the KD decreased body weight, with the alcohol-dependent rats on the KD showing the greatest weight loss (6). The rats in the Dencker et al. (6) study were fed a KD or regular chow *ad libitum*. Therefore, the KD may have been less appetizing or more satiating then the regular chow, which could help to explain the greater weight loss in rats fed that diet. Studies that control for caloric intake are necessary to understand the interaction of KD with alcohol on weight loss.

In a randomized, blinded, placebo-controlled nutritional intervention in inpatients with AUD who were undergoing detoxification, during the first week of withdrawal a KD reduced benzodiazepine use more than a standard diet (50% calories from carbohydrates, 15% calories from protein, and 35% calories from fat) (8). Although withdrawal symptoms measured with the Clinical Institute Withdrawal Assessment—Alcohol revised did not differ between diet groups, patients in the standard American control diet received more benzodiazepines than patients treated with the KD. In the brain, the KD elevated levels of the metabolic markers acetone, AcAc, and glutamate and decreased choline and myo-inositol, metabolites linked to neuroinflammation (8). Correlations between low plasma BHB levels and greater social impairment, depression, and brain white matter alterations in patients with AUD also support the clinical relevance of BHB (43).

Patients who are seeking treatment for AUD often present with poor nutritional status and low appetite (44). Recently, Bornebusch et al. (7) retested the effect of a KD diet on alcohol withdrawal symptoms in mice, which included a KE-treated cohort. In two separate experiments, the researchers tested a “ketosis throughout” cohort, in which ketosis was induced during alcohol administration and abstinence, and a “ketosis after” cohort in which ketosis was induced only during abstinence. The KD diet reduced handling-induced convulsions and anxiety-like behaviors only in the ketosis throughout group, whereas a KE alleviated these withdrawal symptoms in both groups. Moreover, oral administration of 3-hydroxybutyrate alleviated tremor but not muscular rigidity in alcohol-dependent rats (45). This is important because adherence with a KE is greater than that observed with the KD. Although oral D-BHB supplements appear to have a positive therapeutic effect in alleviating withdrawal symptoms in animal models, studies are needed to elucidate the specific symptoms that are reduced and whether oral D-BHB supplements have similar effects on AWS in patients with AUD.

## Effects of Nutritional Ketosis on Alcohol Craving, Consumption and Sensitivity

There is evidence that KD and KE reduce appetite and food intake (15, 46) and rodent studies have shown that nutritional ketosis reduces alcohol intake. Rats maintained on a 9-week KD followed by an 8-week regular chow diet self-administered less alcohol than those with no previous exposure to KD (i.e., mean history of KD = 30.8 ± 4.3 reinforcers/30 min vs. regular Chow = 48.3 ± 6.3) (8). Thus, a history of a KD deescalated alcohol consumption in alcohol-dependent rats (8). Although the authors initially aimed to study alcohol self-administration in rats on a current KD, the large group difference in blood alcohol levels as a function of the KD was a confounder. Specifically, rats on a current KD showed blood alcohol levels that were less than five-fold elevated following alcohol vapor exposure, an effect not seen with a regular chow diet (8). Although this suggests that a KD could interfere with alcohol metabolism, potentially due to altered activity of alcohol dehydrogenase (ADH) or aldehyde dehydrogenase (ALDH) enzymes in the liver, the hypothesis requires testing. Mice exposed to a 7-day KD showed a lower level of alcohol self-administration than those given a standard diet (i.e., mean KD = 0.51 ± 0.04 g/kg vs. standard diet = 1.04 ± 0.08 g/kg) (47). Together, these findings suggest that both current KD and a history of KD lower alcohol consumption in rodent models of alcohol drinking and dependence. However, more research is needed to investigate the effects of a KD on alcohol consumption when differences in blood alcohol levels are accounted for and to assess the effects of a KD and other means of inducing ketosis on acetaldehyde/acetate levels and ADH/ALDH enzyme activity.

We are not aware of human studies that show the effects of nutritional ketosis on alcohol metabolism, tolerance, or consumption. However, in an inpatient clinical trial testing the effects of KD on AWS signs and symptoms during detoxification, 3 weeks of KD were associated with lower subjective ratings of alcohol “wanting” and alcohol craving (at the level of a trend) than an isocaloric standard (control) diet (8). A functional magnetic resonance imaging component of the study also showed that during the 3-week treatment period, there were greater brain dorsal anterior cingulate cortex responses to alcohol visual cues in the KD group than the isocaloric control diet group, which may indicate enhanced control of alcohol craving in the KD group. In individuals with obesity, a 4-month KD lowered food craving and craving for alcohol (46). Interestingly, although alcohol alone did not increase plasma BHB in healthy volunteers, alcohol combined with a KD elevated BHB nearly 8 times more than the KD alone (48). A potential mechanism for these effects could be that elevated acetate concentrations resulting from alcohol catabolism compete with BHB as fuel for the tricarboxylic acid (TCA) cycle, resulting in higher BHB levels in plasma.

Clinical trials are currently underway (NCT04616781; NCT03255031; NCT03878225) that assess the effects of nutritional ketosis on alcohol consumption, metabolism, and tolerance in AUD and to explore potential mechanisms of action of the dietary manipulation.

## Potential Mechanisms of Action for The therapeutic Effects of Ketosis in Alcohol Use Disorder

### Low Glucose Utilization/High Acetate Metabolism

**Figure 1:**
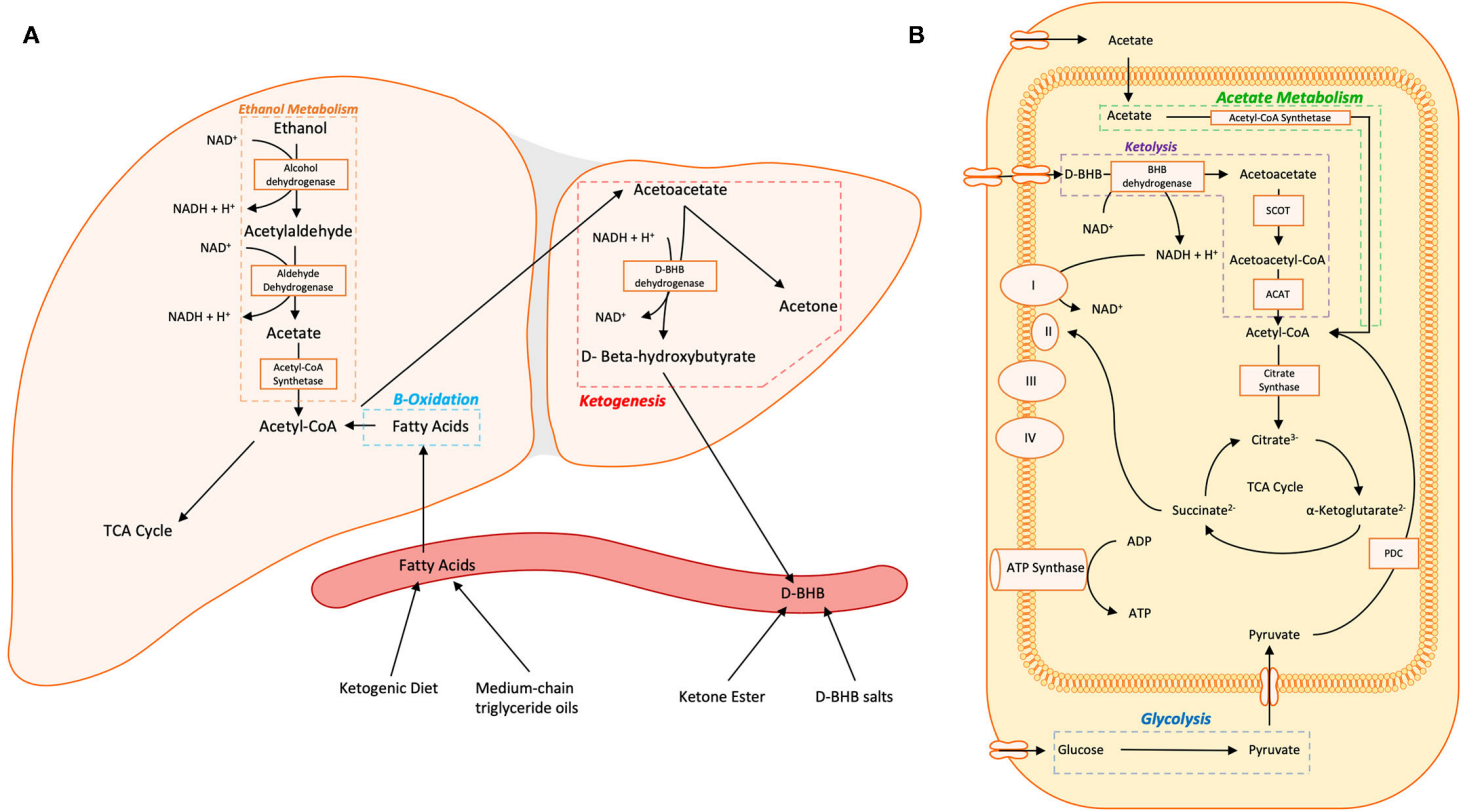
**(A)** Hepatic metabolism of ethanol and fatty acids and ketogenesis are shown. **(B)** Non-hepatic metabolism of D-BHB, acetate, and glucose in the cytosol and mitochondria converge on acetyl-CoA which enters the tricarboxylic acid (TCA) cycle. Within neurons, acetoacetate and D-BHB are transported into the mitochondria *via* monocarboxylate transporters. Abbreviations: TCA Cycle, tricarboxylic acid cycle/the citric acid cycle/Kreb's cycle; D-BHB, D-β-Hydroxybutyrate; NAD(H), nicotinamide adenine dinucleotide; SCOT, 3-ketoacid CoA transferase; ACAT, acetyl-CoA acetyltransferase; I, NADH ubiquinone oxireductase; II, succinate dehydrogenase; III, CoQH_2_-cytochrome c reductase; IV, cytochrome c oxidase; ATP Synthase, F_O_F_1_ ATP synthase; PDC, pyruvate dehydrogenase complex.

Glucose is the brain's primary fuel source in meeting its intensive energy demand. However, temporal variations in the brain's energy demand and supply necessitate alternative additional fuel sources to meet its metabolic and energy challenges. Circulating ketone bodies (AcAc, BHB and acetone) provide metabolic fuel the supply of which can be elevated through carbohydrate fasting-induced hepatic catabolism of fatty acids or exogenous supplementation. Passing through the brain blood barrier and entering the mitochondria of cells in the brain through monocarboxylate transporters, BHB is metabolized into AcAc and then into acetyl-CoA, which feeds into the TCA cycle (Figure 1). Studies of D-BHB supplementation have shown benefits of providing ketones as an alternative to glucose as an energy source for the brain. Some of these benefits include a elevation in the nicotinamide adenine dinucleotide (NAD) redox state (NAD+/NADH) (49, 50), which is important for mitochondrial function, an increase in the free energy for ATP synthesis in neurons (49, 51, 52), and furnishing the cell with acetyl-CoA and citric cycle intermediates (53). These findings lend support to the therapeutic potential of nutritional ketosis in pathologies characterized by glucose insensitivity by providing an alternative energy substrate.

**Figure 2:**
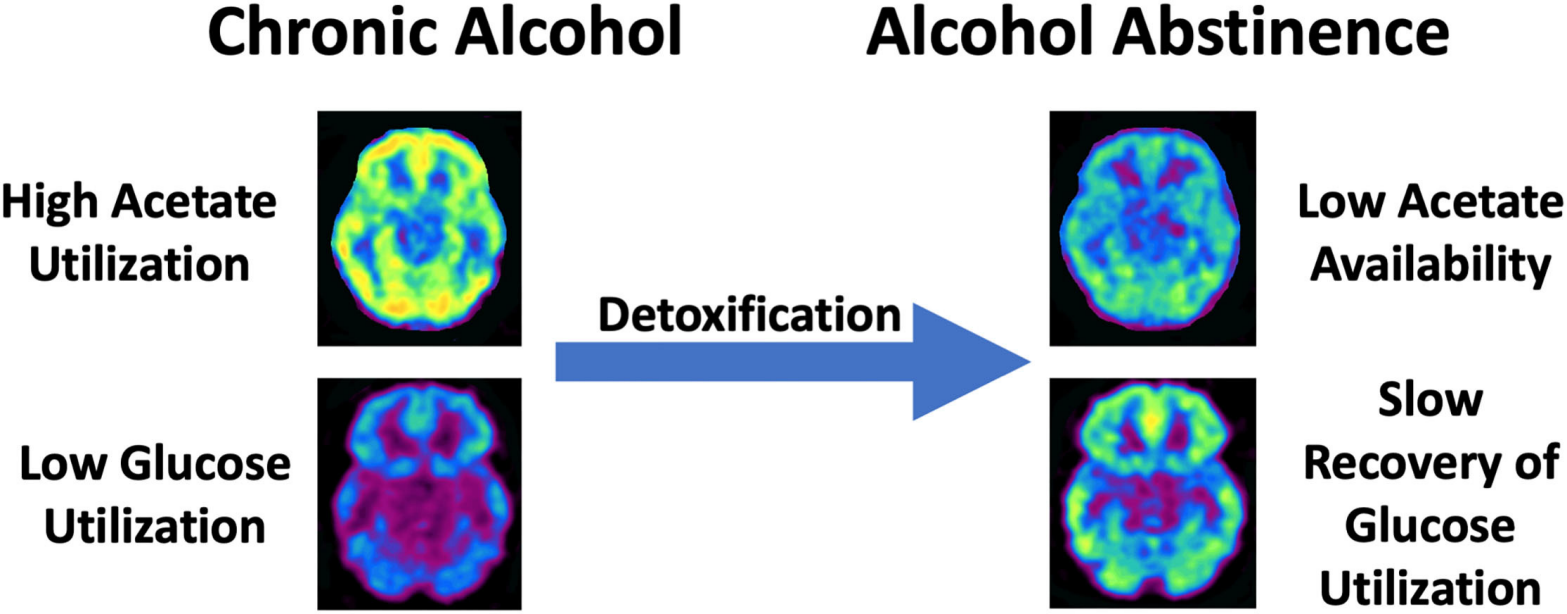
Schematic overview of the shift from high acetate utlization to low brain acetate avaliability with slow recovery of brain glucose metabolism in chronic AUD during detoxification. This shift is hypothesized to produce a central energy deficit that could contribute to alcohol withdrawal symptoms and associated neurotoxicity.

Substantial research has examined the metabolomic and bioenergetic effects of alcohol on the brain. Acute alcohol administration changes the brain's energetics, decreasing glucose metabolism while increasing the metabolism of acetate, a metabolite of alcohol (54). This alcohol-induced shift in brain energetics appears to be accentuated in AUD patients who, during sobriety, show higher brain acetate metabolism (55, 56) and lower brain glucose metabolism (54, 57) than non-alcohol dependent controls. These findings suggest that a shift from glucose to acetate metabolism persists beyond acute intoxication in individuals with AUD (Figure 2). During alcohol detoxification, when acetate supplies are low, this could lead to a central energy deficit that could contribute to the AWS and associated neurotoxicity (56) (Figure 2). The energy substrate deficit can be alleviated by increasing plasma ketone concentrations. Indeed, nutritional ketosis induced by a KD or oral D-BHB (ketone salts) decreased brain glucose metabolism, assessed with fludeoxyglucose ([^18^F]FDG-PET) and increased brain acetate metabolism, with [^11^C]acetoacetate binding in healthy controls (12, 58). However, aging may influence this effect, as Roy et al. (59) showed both elevated brain [^18^F]FDG and [^11^C]acetoacetate in aging rats after KD.

Brain studies in Alzheimer's disease can provide a useful parallel for AUD, as both diseases are associated with reductions in the global cerebral metabolic rate of glucose, which is estimated at 20–25% (60, 61). Reduced glycolytic flux and uptake (62) could help to explain this hypometabolism. Ketogenic diets and ketone supplementation have been shown to be protective in *in vitro* neuronal cell models (63) and benefits in clinical trials of Alzheimer's Disease (37, 64–66). Nutritional ketosis induced by the administration of MCT supplements has been shown to improve memory (67), and to double brain AcAc consumption in individuals with Alzheimer's disease, thereby increasing total brain energy metabolism without affecting brain glucose utilization (68). The relationship between plasma ketones and brain ketone uptake was the same in individuals with Alzheimer's Disease as in healthy young adults (58), indicating that there is intact AcAc utilization in Alzheimer's Disease. Thus, there is a potential for interventions that elevate circulating ketone bodies, primarily the administration of D-BHB, to be useful in treating pathologies characterized by impaired glucose metabolism and supply such as Alzheimer's Disease and AUD.

### Imbalances in Glutamate and GABA

AWS is characterized by a general hyperexcitability of the central nervous system (69). The amino acids glutamate and γ-aminobutyric acid (GABA) are respectively the major excitatory and inhibitory neurotransmitters in the brain. Although alcohol initially inhibits excitatory effects by glutamate transmission and facilitates the inhibitory actions of GABA, chronic alcohol exposure results in compensatory changes in these amino acid transmitter systems that are opposite those seen with acute exposure and may contribute to alcohol withdrawal (70). There have been contradictory findings on brain glutamate concentrations in AUD from proton magnetic resonance spectroscopy (^1^H-MRS) studies. Glutamate levels in the nucleus accumbens (71) and thalamus (72) have been shown to be elevated in individuals with AUD compared to non-dependent controls. However, glutamate levels in the anterior cingulate cortex have been reported to be higher (73), lower (74, 75) or unchanged (71, 72, 76) in AUD individuals during early withdrawal compared to non-dependent controls.

Glutamate in the cingulate of AUD patients was inversely correlated with the number of heavy drinking days in the 14 days preceding the MRS scan (76). Additionally, the number of drinking years but not drinks per day was associated with higher concentrations of glutamate and glutamine (Glx) in AUD. Mon et al. (75) concluded that sobriety may normalize glutamate levels over the course of abstinence. GABA levels in plasma (70) and cingulate cortex (77) have been shown to be low during acute alcohol withdrawal. Moreover, initially low cingulate GABA levels may normalize within 3 days of last alcohol intake but only in treatment-naïve individuals with more severe AUD (77). Thus, more research is needed to better understand the dynamics of brain glutamate and GABA in individuals with AUD.

Glucose metabolism through the TCA cycle is both the main source of energy to the brain and the main source of carbon for the synthesis of glutamate and GABA (78, 79). In a murine model with reduced brain-specific pyruvate dehydrogenase activity, reduced flux through the TCA cycle reduced the glutamate content of the brain and elicited epileptiform discharges, which were ameliorated by acetate administration (80). A recent study in a mouse model of Alzheimer's Disease (triple transgenic Alzheimer's 3xTgAD, which shows reduced brain glucose utilization) showed higher hippocampal glutamate and α-ketoglutarate (a precursor of glutamate) in animals who received a KE diet compared to regular chow and a positive correlation between glutamate and α-ketoglutarate levels in both groups (50). Thus, nutritional ketosis appears to furnish mitochondria with TCA cycle substrates (38), as evidenced by the finding that a 4-month KD elevated glutamate and glutamine in young adult rats (81). Although patients with epilepsy did not show differences from controls in posterior cingulate cortical glutamate measures, patients' elevated glutamate concentrations predicted short-term freedom from seizures, supporting the clinical relevance of glutamate concentrations in epilepsy (82). This underscores the need to elucidate the mechanism(s) underlying the association of KD-induced changes in glutamate with brain excitability.

Vesicular glutamate transporters (VGLUT) are required for packaging and exocytotic release of glutamate. VGLUT is inhibited by AcAc and BHB through a competitive interaction with the VGLUT allosteric activator Cl^−^ (83). A decrease in the concentration of glutamate per vesicle from VGLUT inhibition reduces glutamatergic activation, thereby dampening excitation. BHB and AcAc may also dampen neuronal excitability *via* their effect on K+/ATP channels, having been shown to reduce the spontaneous firing rate of substantia nigra pars reticulata neurons *in vitro* (84). This effect was abolished by the genetic or metabolic elimination of metabolically sensitive K^+^/ATP channels (84). In a recent study, acetone and BHB acted as inhibitors of glutamate at NMDA receptors (85). In addition, D-BHB and acetoacetate reduced neuronal death and changes of neuronal membrane properties in rat neocortical neurons subjected to glutamate excitotoxicity (86). Further, calorically restricted KD increases the expression of glutamic acid decarboxylase, the enzyme responsible for the conversion of glutamate to GABA (87), in the brain which increases the conversion of glutamate and thereby reduces excitation. Thus, there is conflicting evidence and potential mechanistic roles of glutamate in the context of AUD and further elucidation is needed with a particular emphasis on intracellular versus extracellular changes.

The efficacy of a KD in preventing or reducing seizures in epilepsy (88) may have direct relevance to alcohol withdrawal, which can be complicated by seizures. The following mechanism(s) have been proposed for the reduction of seizures by a KD: (1) restoring glutamatergic neurotransmission and enhancement of GABA synthesis, (2) circumventing glycolysis and providing Acetyl-CoA for the TCA cycle through fatty acid oxidation, (3) stimulating ATP-sensitive K^+^ channels, and (4) inhibiting voltage-dependent Ca^2+^ channels (78, 89). However, seizures are uncommon in AUD patients undergoing detoxification, partly because benzodiazepines, which are widely use to manage the AWS, have anticonvulsant activity (90). A KD may reduce overall neuronal excitability, mitigating the severity of alcohol withdrawal symptoms and reducing the need for benzodiazepine treatment during acute withdrawal. Thus, rodent studies are needed to investigate the effect of a KD on alcohol-induced seizures, as these would inform efforts to prevent alcohol withdrawal-induced seizures in patients (90). However, it is unclear how the hypothesized reductions of neuronal excitability with KD would associate with brain glutamate concentrations.

### Hormonal Regulation

Ghrelin is a homeostatic hormone that stimulates human appetite, having effects opposite to those of leptin (91, 92). Endogenous peripheral ghrelin levels decrease during alcohol drinking and increase during alcohol abstinence (93–99). Studies have shown that genetic or metabolic reductions in ghrelin levels decrease alcohol intake (100, 101). In addition, higher ghrelin levels are associated with greater self-reported craving (97, 98, 101, 102), longer and more intense subjective responses to alcohol (103), and activation of the bilateral insulae (104) and ventral striatum (105) as measured with functional magnetic resonance imaging during alcohol cue exposure. Higher levels of ghrelin and activation of the ghrelin receptor stimulate the cholinergic-dopaminergic reward link, which has implications for the reinforcing effects of ghrelin in AUD (106). In healthy volunteers, a single administration of a KE was associated with decreased self-reported hunger and plasma ghrelin levels than the ingestion of isocaloric dextrose (15).

Although there is some indication that KD may suppress ghrelin levels (see review by Roekenes and Martins (107)), there are some inconsistencies in the literature (108–111). Leptin and peptide YY have effects opposite to ghrelin, in that they promote satiety (112, 113). A KD has been shown to increase serum peptide YY levels (114), though it has also been shown to decrease leptin levels (115, 116).

Fibroblast growth factor 21 (FGF21) is a hormone of hepatic origin whose targets include white and brown adipose tissue, the hypothalamus, and the hindbrain (117, 118). A KD has been shown to increase the concentration of FGF21 in murine models (119, 120), but this effect was not seen in humans (121–123). Nevertheless, in humans, FGF21-based pharmacotherapy decreased body weight (124) and variation in the *FGF21* gene has been associated with macronutrient preference (carbohydrate, fat, and protein) (125). Moreover, FGF21 administration reduced a preference for alcohol in mice and for sweets in mice and monkeys (126). Therefore, FGF21 may be a key factor involved in the effects of ketosis on alcohol preference and warrants further investigation.

Glucagon-like peptide 1 (GLP1) is an intestinal hormone that enhances insulin secretion, inhibits glucagon secretion, and decreases gastric motility (127). There is some clinical evidence that the concentration of GLP-1 is increased in response to high fat KDs (109, 128), although experiments in cell culture have yielded contradictory evidence (129). GLP-1 receptor activation by GLP-1 agonists suppresses the effects of alcohol on the mesolimbic dopamine system and decreases alcohol consumption and operant self-administration (130–135). However, the GLP-1 receptor agonist Exendin-4 failed to attenuate morphine conditioned place preference or remifentanil self-administration (132). In addition, there is limited evidence that GLP-1 receptor agonists affect cocaine consumption (136, 137). Taken together, GLP-1 receptor activation induced by increased GLP-1 levels produced by a KD could serve as a suppressor of alcohol intake. Further research is needed to establish the role of the KD effect on circulating GLP-1 levels.

Evidence suggests that alcohol dependence is associated with dysregulation of the hypothalamic-pituitary-adrenal (HPA) axis and extrahypothalamic glucocorticoid signaling as well as other stress (e.g., corticotropin-releasing factor [CRF]) and anti-stress (e.g., neuropeptide Y) systems (138). However, the few available studies of the effects of ketosis on the HPA axis and other stress systems have yielded contradictory findings. For example, in one rat study, neither a KD nor a ketone supplementation diet affected plasma levels of adrenocorticotropic hormone or corticosterone (139). In another study, both KD and MCT increased HPA axis activity (140). Interestingly, in female but not male rats exposed to chronic mild stress, a KD prevented stress-related blood corticosterone and hypothalamic NPY expression; this effect was not accompanied by altered CRF mRNA expression (141). Furthermore, continuous microinjection of D-BHB into the prefrontal cortex attenuated the effects of a chronic unpredictable stress on depression-like behavior and HPA axis activity (142). More research on the effects of ketosis on stress systems is needed.

### Nicotinamide Adenine Dinucleotide (NAD+)

NAD^+^ is present in all living cells and plays a vital role in cellular metabolism as a coenzyme for redox reactions, including those required for mitochondrial energy production. NAD^+^ decreases with age (143, 144) and lower NAD^+^ levels are associated with neurodegenerative and neuropsychiatric disorders including Alzheimer's Disease and schizophrenia (145). Although individuals with AUD have low liver concentrations of NAD^+^ (146), it remains to be determined whether their brain NAD^+^ concentrations are affected by chronic heavy alcohol consumption. Because NAD^+^ and pyruvate are implicated in both the oxidation of alcohol (147, 148) and in the metabolic effects of fasting (149), it is possible that these compounds mediate the clinical efficacy of nutritional ketosis in AUD. An intravenous infusion of NAD+ during alcohol or opioid withdrawal attenuated both craving and withdrawal symptoms (150). A 7-Tesla magnetic resonance spectroscopy study in healthy volunteers showed that ketone supplementation elevates the concentration of NAD^+^ in the brain (151). Mice who received dietary supplementation with KE had higher cortical and hippocampal free cytosolic [NAD^+^]/[NADH] than mice fed a control diet (50). A KD also increased cellular concentrations of NAD^+^ (152), along with concentrations of Sirt1, Parp-1, and 8-hydroxy-2′-deoxyguanosine, which could improve brain health by increasing resilience to DNA damage and oxidative stress (153). However, the effects of ketone modulation of NAD^+^ in patients with AUD and its clinical and cognitive effects are unstudied. Because nucleotide coenzymes and their corresponding oxidizing forms are compartmentalized and bind at a subcellular level, their measurement and the interpretation of the results require great care to ensure accuracy. For example, from fed, freeze-clamped, rat liver the calculated free cytoplasmic [NAD^+^]/[NADH] from lactate dehydrogenase was approximately 200 times higher than the ratio calculated measured using total concentrations of the coenzymes. Conversely, the free cytoplasmic [NADP^+^]/[NADPH] from isocitrate dehydrogenase was ~20 times lower than the ratio calculated from measured total respective amounts (149).

### D-β-Hydroxybutyrate as a Signaling Molecule

In addition to its direct action in mitochondrial metabolism, D-BHB may exert therapeutic effects as a signaling molecule. D-BHB has been suggested to have direct involvement in epigenetic regulation due to its ability to act as an inhibitor of class 1 histone deacetylases (HDAC) that increases global acetylation levels in a dose-dependent manner (154). During withdrawal from chronic alcohol, anxiety-like behaviors were correlated with an increase in HDAC activity and a decrease in H3/H4 acetylation, but the behaviors could be reversed with the HDAC inhibitor trichostatin A (155). Furthermore, alcohol withdrawal-induced hyperalgesia was attenuated by the HDAC inhibitor suberoylanilide hydroxamic acid (156). In addition, the anxiolytic-like responses to acute alcohol administration were associated with increased histone acetylation and HDAC inhibition in the amygdala (155, 157). *In vitro* application of D-BHB also increased the expression of *FOXO3, MnSOD, CAT*, and *MT2*, genes that encode oxidative stress resistance factors (154). Bolstering this mechanism, various studies have demonstrated D-BHB's neuroprotective effect against oxidative stress (158–160). In humans, alcohol is processed by ADH enzymes into acetaldehyde, which produces unstable free radicals like hydrogen peroxide and superoxide (161, 162). Furthermore, chronic alcohol consumption depletes mitochondrial glutathione, a potent antioxidant (163). Thus, D-BHB may be unique in its capacity to respond to epigenetic and oxidative stress changes that occur during AUD.

Brain-derived neurotrophic factor (BDNF), a neurotrophin that helps control neurogenesis, has been implicated in the development of AUD (164–166). Although individuals with current AUD had lower overall serum BDNF levels than non-AUD controls (167), preclinical studies indicate that the directionality of the BDNF change is brain region-specific (168, 169). Further, BDNF levels raise during alcohol withdrawal in preclinical models (170), clinical populations (171–173), and raise during withdrawal from other addictive drugs (174, 175). Mechanistically, D-BHB enhances the expression of BDNF through downstream targeting of CREB and acetylation of *BDNF* promoters (176–178). While some clinical evidence points to serum BDNF being significantly increased following adherence to a KD (116, 179, 180), Vizuete et al. (181) found a KD decreased striatal BDNF levels and had no effect on hippocampal levels of BDNF in Wistar rats. The KD and D-BHB's effect on BDNF expression in the context of AUD warrants investigation.

D-BHB is a ligand of the hydroxyl carboxylic acid receptor type 2 (Hca2) (182), a GPCR encoded by the *Hcar2* gene that mediates anti-inflammatory effects (183). In a rodent stroke model, a KD and D-BHB separately rescued stroke-induced neurological deficits but the effect was not seen in *Hcar2* knockout mice (Hcar2^−/−^; (184). These findings reinforce the critical role of Hca2 as an intermediate for D-BHB's neuroprotective effects. In addition to lower hepatic D-BHB levels in humans with alcohol-associated hepatitis, D-BHB attenuated abnormalities in plasma ALT levels, steatosis, and hepatic trigylceride levels induced by the β-oxidation inhibitor etomoxir and alcohol (185). The protective effect of D-BHB was not seen in *Hcar2*^−/−^ mice (185).

The NLR family pyrin domain containing 3 (NLRP3) inflammasome complex is a predominately macrophagic protein that mediates caspase-1 activation and the secretion of proinflammatory cytokines in response to mitochondrial dysfunction, ROS and more. Evidence suggests that the NLRP3 inflammasome complex is activated by alcohol consumption (186, 187) and inhibited by D-BHB supplementation (188). Although deficiencies of NLRP3 were shown to attenuate alcohol-associated steatosis (189), a study showed that this inhibition can increase the rate of hepatic damage (190), suggesting that the NLRP3 inflammasome complex may be protective during alcohol-induced hepatic damage. *In vitro* inhibition of the NLRP3 inflammasome by D-BHB was decreased by high insulin or high glucose, suggesting an influence of the metabolic state of the cell (191). Finally, a single dose of D-BHB clinically was shown to increase markers of NLRP3 inflammasome activation blood cells (192); however, this finding failed subsequent replication in patients with obesity (193). Further research is needed to elucidate the role of metabolic ketosis on the NLRP3 inflammasome in the context of AUD.

## Conclusion and Future Directions

Preclinical and clinical research on the role of ketosis in the signs and symptoms of the AUD/AWS suggest that such an intervention could be useful as an adjunctive treatment. We reviewed potential mechanisms of clinical action of ketosis, with a particular emphasis on brain energy substrate utilization and the glutamatergic/GABAergic systems. An existing limitation of the proposed therapy is the potential of a KD to contribute to the development of alcohol-associated ketoacidosis, which occurs with some frequency in patients with AUD. Thus, clinical trials of ketosis as a treatment may need to exclude participants at increased risk of ketoacidosis and a history of ketoacidosis. Younger patients may be more susceptible to symptomatic hypoglycemia following adherence to a KD (194). The ingestion of ketone ester can also decrease blood glucose concentrations (14, 139). In addition, sex differences in alcohol metabolism and in the response to a KD warrant investigation (195–197), as does variation in genes involved in alcohol and fat metabolism (e.g., *ADH, ALDH, FGF21*) (120, 198–200). The existing literature supports further examination of nutritional ketosis as a therapeutic target for AWS and of the mechanistic underpinnings of its effects. Moreover, key questions as to the effects of nutritional ketosis on brain energetics in AWS, alcohol tolerance, and AUD-associated brain hypometabolism remain to be investigated.

## Author Contributions

VM and CW drafted the first version of the manuscript. SE, LV, GK, VD, MK, HK, and NV provided critical input that significantly improved the manuscript. All authors contributed to the article and approved the submitted version.

## Funding

LV was supported by NIDA Intramural Research Program. HK is a member of an advisory board for Dicerna Pharmaceuticals and Sophrosyne Pharmaceuticals, a consultant for Sobrera Pharmaceuticals, and a member of the American Society of Clinical Psychopharmacology's Alcohol Clinical Trials Initiative, which was supported in the last three years by AbbVie, Alkermes, Dicerna, Ethypharm, Indivior, Lilly, Lundbeck, Otsuka, Pfizer, Arbor, and Amygdala Neurosciences. CW was supported by a K99/R00 Pathway to Independence Award (AA026892) and a NARSAD Young Investigator Grant (28778), Brain & Behavior Research Foundation.

## Conflict of Interest

HK is named as an inventor on PCT patent application #15/878,640 entitled: Genotypeguided dosing of opioid agonists, filed January 24, 2018. The remaining authors declare that the research was conducted in the absence of any commercial or financial relationships that could be construed as a potential conflict of interest.

## Publisher's Note

All claims expressed in this article are solely those of the authors and do not necessarily represent those of their affiliated organizations, or those of the publisher, the editors and the reviewers. Any product that may be evaluated in this article, or claim that may be made by its manufacturer, is not guaranteed or endorsed by the publisher.
